# Post-therapeutic support and rehabilitation for breast cancer women in complete remission in a thermal resort: study protocol for the PACThe Real-Life conditions observational trial

**DOI:** 10.1186/s12885-025-13524-2

**Published:** 2025-01-29

**Authors:** Mélanie Godiveau, Judith Passildas, Emilie Thivat, Mélanie Casile, Céleste Pinard, Alexia Giro, Marie-Ange Mouret-Reynier, Ioana Molnar, Catherine Abrial, Claude-Eugène Bouvier, Yves-Jean Bignon, Xavier Durando

**Affiliations:** 1 Division de la Recherche Clinique, Centre Jean PERRIN, 58 rue Montalembert, Clermont-Ferrand, 63011 France; 2 Collège des Economistes de la Santé, Villejuif, France

**Keywords:** Breast cancer, Health-related quality of life, Post-cancer rehabilitation, Thermal therapy

## Abstract

**Background:**

Over the past twenty years, the post-cancer rehabilitation has been developed, usually in a hospital setting. Although this allows better care organization and improved security, it is perceived as stressful and restrictive by the “cancer survivor”. Therefore, the transfer of benefits to everyday life is more difficult, or even uncertain. Staying in a thermal resort seemed to offer a good compromise: the security and structure of centralized reception in a thermal resort for treatment and medical supervision, and accommodation in a hotel with full dietary board. It combines institutional quality and security with the freedom of real life and the dynamics of a group formed under innovative conditions. This rehabilitation procedure was tested with the program PACThe (Program of Accompanying women after breast Cancer treatment completion in Thermal resorts) which was evaluated at one year in a randomized controlled trial involving 251 patients [1] and at five years in another study [2]. The results shown a significative improvement in many areas such as quality of life, functional abilities and sleep. The aim of this study is therefore to confirm the benefits of the PACThe program at six months for breast cancer women in complete remission in real-life conditions.

**Methods:**

The project PACThe Real-Life conditions is an observational trial in which 400 breast cancer women in complete remission will be sent to a 3 week thermal “post-cancer rehabilitation” program. Primary objective is to assess the quality of life until 12 months. The outcome will be measured with the self-questionnaire SF-36 [3] at five timepoints (inclusion visit, end of thermal resort, 6 months post-cure, 12 months post-cure and 18 months post-cure). The duration of patient participation in the study will therefore be 18 months. The main analysis will consist of comparing the SF-36 global score at 6 months with the baseline.

**Discussion:**

This study aims to investigate the improvements in quality of life of 3 weeks stay at thermal resort for breast cancer women in complete remission. The comprehensive overview of the program PACThe will likely contribute to enhancing thermal therapy post-cancer rehabilitation in this population in real life conditions.

**Trial registration:**

The PACThe Real-Life conditions trial is a prospective observational study registered at the ANSM registry (2022-A00171-42) on March 1, 2022. This study has been registered on ClinicalTrials.gov: NCT05433077. Protocol: version 4, 22 December 2022.

## Background and aims of the study

As a result of early detection methods and increasingly effective treatments, more and more patients are surviving breast cancer. For instance, according to the World Health Organization, in 2022, the 5-year survival rates for breast cancer in western Europe exceeded 90% [4]. Therefore, improving the quality of life after treatment is a major challenge for oncologists.

Breast cancer patients often experience a significant decline in their quality of life (QoL) after surgery, chemotherapy, and radiotherapy which can lead to depression symptoms in the months following treatment.

Physical capacity constitutes a fundamental component of overall QoL, but cancer treatments frequently reduce physical capabilities, often due to surgical complications and metabolic changes resulting from chemotherapy, causing weight gain. The NHANES study shows that a significant portion of survivors spend most of their day in sedentary or low-intensity activities, leading to higher body mass index (BMI) and decreased physical QoL [5]. Additionally, post-breast cancer physical activity has an impact on survival, with a meta-analysis showing a significant reduction in mortality [6], and has been linked to better overall health [7].

However, encouraging cancer survivors to engage in physical activity remains a difficult task due to significant exercise barriers, such as fatigue, that impede their participation and commitment to cancer rehabilitation programs [8]. This is why participation in a specific rehabilitation program had to be carefully considered.

Post-cancer rehabilitation has been developed over the last twenty years. Most programs are implemented in hospital settings, which ensure the best possible safety and organization of care, but are perceived as restrictive and stressful for the “cancer survivor” after long months of heavy treatment; in addition, the transfer of benefits to everyday life is more difficult, if not uncertain. Outpatient treatment is less prone to these drawbacks, but limits access to people living in sufficiently large urban areas.

Staying in a thermal resort seemed to offer a good compromise: the security and structure of centralized reception in a thermal establishment for treatment and medical supervision, and accommodation in a hotel with full dietary board. It combines institutional quality and security with the freedom of real life and the dynamics of a group formed under innovative conditions.

Traditionally, programs in thermal resorts have been contraindicated for cancer patients during progression. However, during periods of complete remission, thermal programs can be a beneficial complementary therapy [9].

The “PACThe” program (Program of Accompanying women after breast Cancer treatment completion in Thermal resorts) enabled women treated for breast cancer (severe forms having received chemotherapy, surgery, radiotherapy) and in complete remission to be cared for in thermal establishments on a full-board dietary basis. The objectives of the program were to improve the patients’ quality of life by restoring their functional capacities, ensuring efficient weight control, combating stress and compensating for social and socio-aesthetic handicaps. To this end, the program combined hydrothermal balneotherapy, adapted physical activity, nutritional and dietary education, socio-aesthetic advice and psychological follow-up.

The program’s impact was evaluated at one year in a randomized controlled trial involving 251 patients (number calculated according to the assigned objective of improvement in the primary endpoint) [1]. This trial was complemented by an ancillary medico-economic study that was published separately [10]. A third study assessed quality of life (the study’s primary endpoint) at five years in surviving patients [2].

The results at one year show that implementation of the program is statistically significantly associated with an improvement in the following elements: quality of life measured with the SF36 QoL scores [3] at 6 and 12 months and compared between the “SPA group” and the “control group”; functional capabilities; weight control, particularly in overweight patients; depressive situation; sleep; level of physical activity in previously active patients; and return to work [1].

The intervention proved to be cost-effective at one year for all program participants and at six months for women who had returned to work. The main difference was in the use of physiotherapy, which was higher for patients in the control group [10].

At five years, the significant difference in quality of life was maintained between the two groups (*p* =.009 with a delta of 7.2 in the SF36 QoL score [3]), while mortality rates did not differ.

On the strength of all these results, the post-breast cancer rehabilitation program carried out as part of a stay at a thermal resort and implementing the program evaluated by the PACThe study has received financial support from the French health insurance scheme.

The study “PACThe program in real life” is a real-life trial to evaluate the post-breast cancer rehabilitation program PACThe. The thermal resort setting ensures the safety and availability of care, while remaining closer to real-life conditions than a stay in a healthcare facility.

The direct benefit for patients is a potential improvement in quality of life, the aim of the study being to show a lasting improvement in quality of life following the proposed program. At first sight, there are no foreseeable risks for patients.

The primary aim of the study is to validate that the beneficial results on the quality-of-life score (SF36) of the PACThe study so it could be extended to all women in post breast cancer (medically attested complete remission) regardless of their treatment or the time between the end of their treatment and the start of their thermal program.

Secondary aims are to study the effectiveness of the PACThe program in real life, according to subgroups, and to compare the effects observed (at different time points) with those observed in the PACThe study at the same time points.

## Design and methods

### Design

The study design is a longitudinal cohort observational study within an approved post-therapeutic support and rehabilitation program.

### Coordination and participating institutions

The Conseil National des Etablissements Thermaux (CNETh) is the sponsor. The study will be conducted under the responsibility of Pr Xavier DURANDO, study coordinator. The Clinical Research Division of the Centre Jean PERRIN will be in charge of coordination, submission, data entry, monitoring and statistical analysis.

The list of the study sites is available on https://clinicaltrials.gov/study/NCT05433077.

### Objectives and outcomes

All outcome measures will be assessed at baseline and at 6 months (i.e., after concluding the intervention period).

#### Primary objective and outcome measure

The primary objective is to confirm the improvement in the quality of life at 6 months following the implementation of a post-therapeutic support and rehabilitation program for women in complete remission from breast cancer in thermal resorts (PACThe program), in real-life conditions, regardless of their treatment or the interval between the end of treatment and the start of the program.

The primary outcomes is the change in SF-36 global score [3] between baseline and at 6 months.

#### Secondary objectives and outcomes measure

The secondary objectives are to assess the effectiveness of the PACThe program in real-life conditions based on subgroups and to compare the observed effects (at various time points) with those observed in the original PACThe study at the same time points.

Secondary endpoints will be assessed with the SF-36 questionnaire global and subscale scores obtained at inclusion, end of program, 6, 12 and 18 months after end of program. Scores in subgroups (according to BMI, age, menopausal status, level of education, precariousness (assessed via EPICES score)) will also be assessed.

### Setting and eligibility criteria

Participants have been recruited through referral from French physicians prescribing thermal treatments (among participating centers). The thermal resorts also promote the program in their brochures and on their websites, so that patients and doctors are aware of it. Eligible participants are women aged 18 to 75 who have undergone breast cancer treatments such as chemo- and/or radiotherapy, in the absence of residual or progressive disease, and whose treatment has been completed (with the exception of hormone therapy). The exclusion criteria are defined as follows:

- Progressive or metastatic cancer.
- Patient suffering from disability.
- Severe personality or eating disorders (craving, bulimia, etc.)
- Underweight (BMI < 18.5 kg.m-2) or severe to massive obesity (BMI > 35 kg.m-2).
- Refusal to participate.
- Contraindication to physical activity (whether cardiovascular or related to musculoskeletal pathology); lymphedema is not a contraindication.
- Participation in another clinical study.
- Insufficient knowledge or understanding of the French language to correctly complete a quality-of-life self-questionnaire or to answer a dietary questionnaire.
- The medical contraindications to thermal treatment validated by the Société Française d’Hydrologie et de Climatologie will be applied and are as follows:
- Severe alterations in general condition (progressive infectious conditions, renal or hepatic insufficiency, cirrhosis, respiratory insufficiency, etc.)
- Severe or acute hemorrhagic rectocolitis and Crohn’s disease.
- Severe immunodeficiencies.
- Recent phlebitis.
- Hypertension.

### Intervention and participant timeline

This study is based on the PACThe program [1] which consisted in a two-week follow-up care protocol of 13 days of intervention, averaging 4 sessions per day, along with all the required dietary meals each day. The interventions are defined as physical activity with endurance, muscle strengthening, and flexibility exercises and with aqua aerobic sessions. The participants were also provided with psychological support and self-esteem guidance.

At the start of the stay at thermal resorts and before the first treatment, selection criteria will be checked by the thermal doctor, who also provides initial patient information. After receiving and reading the information notice, the patient gives the signed non-opposition notice to the thermal doctor and keeps a copy before any study procedure. The thermal doctor collects the clinical and medical information required for the program (age, BMI, anti-cancer treatments received, surgery, menopausal status, precariousness, level of education) and enters it into the eCRF. The SF-36 questionnaire is given to the patient. A check-up at the start of the treatment is carried out by one of the health professionals on the program team.

At the end of the stay at the thermal resort, the doctor collects the clinical and medical information required for the program. The thermal doctor gives the patient the SF-36 questionnaire to fill in.

For the follow up at 6-12-18 months, the questionnaires are sent to the patient to be completed.


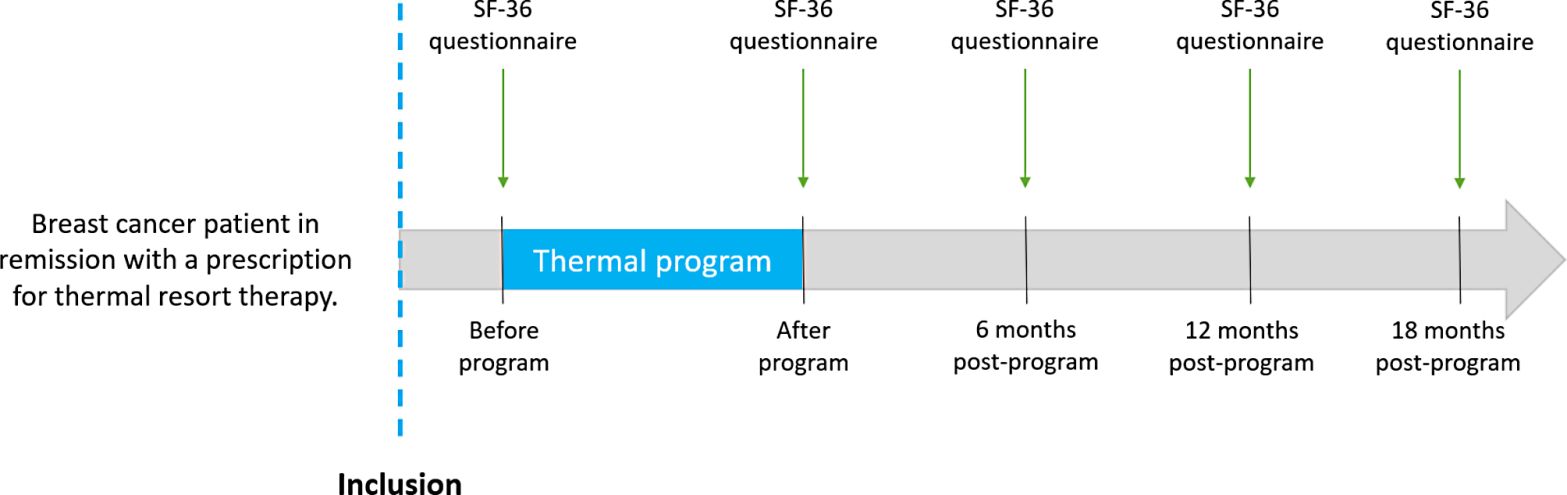


### Sample size

The main analysis consists in comparing the change in SF-36 global score at 6 months from baseline to the value obtained in the PACThe study control group (historical comparator). In the randomized PACThe study, the difference in SF-36 global score between the experimental and control groups was 2.2 points at baseline (*p* =.3), and 9.5 points at 6 months (effect size = 0.63, *p* <.001), with a difference between 6 months and baseline of 11.9 points in the experimental group, and 4.6 in the control group, and a standard deviation between 15 and 16.

To determine the number of subjects required, it is assumed that the variation between 6 months and inclusion in this study will be slightly lower than in the PACThe study: expected value of 10 points. It is also estimated that the variability of the scores obtained will be greater than that observed in the PACThe study. Thus, a sample size of 400 should allow us to show a reasonable effect size of 0.2 (alpha risk equal to 5% and 95% power, for a one-sample Student’s t-test), and compensate for an estimated 15–20% drop-out rate.

### Statistical analysis

The main analysis consists of comparing the change in SF-36 global score [3] between 6 months and baseline with the value obtained in the PACThe control group (historical comparator) using a Student’s t-test (or a non-parametric test depending on the data distribution). All patients who will not respond to the questionnaires at 6 months will be excluded from the analysis.

The evolution of the SF-36 global score over time (repeated data) will be analysed using mixed-effects linear regression models, followed, in the event of a statistically significant result, by post-hoc pairwise comparisons at different time points, with correction for multiple comparisons).

Confidence interval estimates of observed effects (changes in measurements at different time points post-program vs. baseline) will be used for comparison with the effects observed in the PACThe study, as well as analysis in subgroups defined by age, menopausal status and type of anti-cancer treatment.

### Data management and monitoring

All data collected for the study (whether identifiable or not) will be pseudonymized and recorded in an eCRF (Ennov Clinical Software).

Data collection and entry are carried out by the thermal physician (inclusion) and the CRA (Clinical Research Assistant).

All clinical and medical data will be collected directly during the consultations, using questionnaires (quality of life and precariousness score), which will be transcribed on paper and used as a source document.

The collected data will be limited to the bare minimum necessary to achieve the study’s objectives, including clinical information regarding previous cancer treatments (chemotherapy, immunotherapy, radiotherapy, hormone therapy) obtained from the patient during the inclusion visit, relevant clinical factors for program management (age, BMI, menopausal status, socio-economic status (questionnaire), education level (questionnaire)) gathered from the patient during the inclusion visit, and quality of life assessment using the SF-36 questionnaire.

Data circulation is centripetal, converging towards the clinical research department of the Centre Jean Perrin, and consequently towards the coordinator working in the same establishment. In the context of this study, the identifying and sensitive data collected will be general identification data (age, sex, patient code and department of residence), administrative identification data (surname/first name, postal and telephone contact details), health data (weight, height, therapy followed) and other types of data such as socio-professional category, family situation, level of education and lifestyle habits. All data will be pseudonymized and treated as strictly confidential. Data pseudonymization involves assigning a patient code to each patient taking part in the study. Thus, the data entered in the eCRF will not be used to identify patients. A correspondence table will be kept in each thermal resort, enabling the patient code to be linked to the patient’s identity. The only identifying documents will be the chart and the non-opposition form, which will be kept securely (locked cabinet or password-protected computer file).

Data will therefore remain on French territory at all stages. There are no plans to transfer data outside France.

Data will be remotely monitored regularly based on the status of the patients enrolled at the participating center. The monitoring will be performed by a clinical research associate mandated by the Coordonator. The objectives will be to ensure the proper conduct of the study in each center, and the recording of the data generated. The monitoring reports will ensure traceability.

Missing data will not be replaced (they will be managed through partial exclusion). In cases where there is a significant amount of missing data, an analysis will be conducted to assess their statistical nature and potential impact on the results. If deemed relevant, an appropriate imputation method will be suggested.

### Trial status

The PACThe– Real life conditions is currently recruiting. Patient enrolment began in June 2022 and is expected to end in May 2024.

## Discussion

This study will examine the improvement of quality of life at six months after a 3 week post-therapy rehabilitation program in thermal resorts for breast cancer women in complete remission (PACThe program) in real life (regardless of their treatment or the time between the end of treatment and the start of the program). The program PACThe has demonstrated that rehabilitation program based on a stay at thermal resort allowed a significative improvement of the quality of life, functional capacities, depressive situations, sleep, weight control, level of physical activity in previously active patients, and the return to work. This study aims at validating those effects in real life conditions by comparing the experimental group to the control group used in the PACThe program. A number of relevant publications have demonstrated that thermal therapy can complement conventional therapies for rheumatic diseases [11], and it was also described through a review that thermal therapy, hydrotherapy and balneotherapy have positive effects on sleep quality [12]. However, description of that kind of rehabilitation program for cancer survivors has been very poor. Even though only women will be enrolled, this trial will determine the extent to which a post-cancer thermal therapy improves the way patients go back to their life after their remission. Real-life studies offer the advantage of reflecting current clinical practices, allowing to evaluate how treatments perform in real clinical settings. These studies have less strict criteria for patient selection, which means they include a more diverse group of patients, resembling the variety of people seen in real-life clinical settings. Therefore, the results of this observational trial will likely be useful for the development of such care pathway for breast cancer survivors, but also for other cancer survivors.

## Data Availability

Not applicable. The results will be published and will be posted on ClinicalTrial.gov.
